# Comparison of cytotoxicity of black phosphorus nanosheets in different types of fibroblasts

**DOI:** 10.1186/s40824-019-0174-x

**Published:** 2019-11-29

**Authors:** Su-Jin Song, Iruthayapandi Selestin Raja, Yu Bin Lee, Moon Sung Kang, Hee Jung Seo, Hyun Uk Lee, Dong-Wook Han

**Affiliations:** 1 Department of Cogno-Mechatronics Engineering, College of Nanoscience & Nanotechnology, Pusan National University, Busan, 46241 South Korea; 2 Monocrystalline Bank Research Institute, Pusan National University, Busan, 46241 South Korea; 3 Advanced Nano-surface Research Group, Korea Basic Science Institute (KBSI), Daejeon, 34133 South Korea

**Keywords:** Black phosphorus nanosheets, Fibroblasts, Cytotoxicity, LDH assay

## Abstract

**Background:**

Two-dimensional black phosphorus nanosheets (BPNSs) have recently emerged as a successive novel nanomaterial owing to their uniqueness in optical and electrical properties. Although BPNSs have found a wide range of biomedical applications, their biosafety is still a major concern to be addressed.

**Methods:**

In this study, we have prepared layered BPNSs using liquid exfoliation procedure, and evaluated their physicochemical properties using Fourier Transform-infrared (FTIR) spectroscopy, Raman spectroscopy, atomic force microscopy, and Zetasizer analyses. We have investigated potential cytotoxicity of BPNSs against three different types of fibroblast cells, i.e. mouse embryonic fibroblast (NIH3T3), primary cultured normal human dermal fibroblast (nHDF), and fibrosarcoma (HT1080). Cell counting kit-8 (CCK-8) assay was carried out to assess cellular metabolic activity in cells whereas lactate dehydrogenase (LDH) activity assay was helpful to study plasma membrane integrity.

**Results:**

Our salient research findings showed that BPNSs were polydispersed in solution due to aggregation. Toxic response of BPNSs against fibroblast cells was in the order, HT1080>nHDF>NIH3T3. The nanosheets reduced the number of cancerous cells with significant difference to normal cells.

**Conclusions:**

We suggest that BPNSs can be considered for cancer treatment as they destroy cancerous cells effectively. However, a comprehensive study is required to elucidate other biological effects of BPNSs.

## Background

Over the last few decades, the development of nanoscience and nanotechnology has made dramatic progress in research fields towards the understanding of nanomaterials and the production of effective nanomaterials at large scale. Recently, black phosphorus nanosheets (BPNSs) have got attention among the researchers due to their outstanding performances in various areas including electronics, photonics, energy storage, and nanomedicine [1, 2]. They have been promising candidates in biomedical fields such as drug delivery, bioimaging, biosensor, and tissue regeneration [3, 4]. BPNSs loaded cisplatin and oxaliplatin agents were studied for in vitro evaluation of targeted drug delivery, which led to efficacy in cancer treatment [5]. An excellent near-infrared (NIR)-photoresponsive composite was fabricated with shape memory performance and biodegradability using piperazine-based polyurethane and BPNSs. The composite enabled the development of smart implantable devices, which can be controlled by the remote NIR light to alter its functions in the body [6]. However, it is of paramount importance to evaluate the toxicity profile of BPNSs to achieve numerous effective nanomaterials with biocompatibility.

BPNS is composed of only one element with six-membered rings bound together by van der Waals force. It was reported that the elemental atoms in BPNSs are not connected by sp^2^-type bonding and each sheet is uniquely puckered by two chemically bonded double layers [1, 7, 8]. Besides the 2D layered structure, ultrafine black phosphorus quantum dots were also reported to exhibit unique electronic and optical properties owing to the quantum confinement and edge effects. A flexible memory device exhibiting a nonvolatile rewritable memory effect with a high ON/OFF ratio was fabricated by mixing black phosphorus quantum dots with polyvinylpyrrolidone. The quantum dots prepared by a facile top-down approach possessed a lateral size of 4.9 ± 1.6 nm and a thickness of 1.9 ± 0.9 nm [9]. Biodegradable BPQDs/PLGA nanospheres were prepared by loading black phosphorus quantum dots (BPQDs) into poly(lactic-co-glycolic acid) (PLGA) by an oil-in-water emulsion solvent evaporation emulsion method. The in vitro and in vivo characterizations demonstrated that the BPQDs/PLGA nanospheres were biocompatible proving excellent photothermal therapy efficiency and tumor targeting ability [10].

The other known allotropes of phosphorus are white and red phosphorus. Though white phosphorus has been reported to be toxic like cyanide, red phosphorus has been found safe for human beings. The chronic exposure of white phosphorus induced several ailments such as bone necrosis, upper respiratory tract irritation, nasal discharge, and headache [8, 11]. Some research findings showed that BPNSs with small thickness and size have high reactivity towards oxygen and water and degrades in an aqueous medium. Further, the final degradation products are phosphate and phosphonate, which are found nontoxic in the human body [10]. Meanwhile, few literature are available to demonstrate the toxicity profile of black phosphorus with convincing evidences. The cytotoxicity of BPNSs to human lung carcinoma epithelial cells (A549) was investigated after 24 h exposure of cells at different concentrated nanomaterials, i.e. 0–400 μg/mL. Cell viability measurements such as water-soluble tetrazolium salt (WST-8) and methyl thiazolyl diphenyl tetrazolium bromide (MTT) assays revealed that BPNSs reduced cell viability in a dose-dependent manner. At 50 μg/mL of nanoparticles, the percentage of cell viability was 48 and 34% for WST-8 and MTT assays, respectively [8]. In another study, in vitro cytotoxicity measurements of BPQDs revealed that the nanoparticles exhibited significant apoptotic effects on HeLa cells at a high concentration of 200 μg/mL. The in vivo investigations in BPQDs exposed mice indicated that the nanoparticles could induce oxidative stress, DNA damage, and reduction of catalase activity transiently but recover to healthy state gradually [12].

In the current study, we have prepared BPNSs using a modified ultra-sonication-assisted solution method and characterized the sample using FTIR, AFM, and Zetasizer measurements. We have demonstrated cytotoxicity of BPNSs exposing three different types of cells i.e., mouse embryonic fibroblast (NIH3T3), primary cultured normal human dermal fibroblast (nHDF), and human fibrosarcoma cells (HT1080). The experiment was carried out at various concentrations of nanosheets from 0 to 125 μg/mL and at different time 24 h and 48 h. Also, we have investigated enzymatic activity and membrane damage in cells to assess the toxic level by CCK-8 and LDH assays. In brief, the work aims to carry out a preliminary toxicity study to show the possible hazardous effects of BPNSs before taking them for a wide range of biomedical applications in the future.

## Methods

### Fabrication of black phosphorus nanosheets

BPNSs were prepared from the bulk black phosphorus material using liquid exfoliation procedure, as described in previous literature [3]. In brief, 0.4 g of BP nanomaterial was dispersed in a 100 mL of distilled water and subsequently, ultrasound sonication was carried out at 20 kHz frequency for 30 min. The resulting several-layered BPNSs were diluted to 10 times and ultrasonication was repeated until they produce small-sized nanosheets. The fine powder of BPNSs was obtained after drying the sample in open air for 24 h. Before each characterization, the sample was agitated by ultra-sonication for 15 min in distilled water to avoid agglomeration.

### Characterization of BPNSs

The physicochemical properties of BPNSs were evaluated using different characterizations including Fourier transform-infrared spectrum (FT-IR, Nicolet Co., Madison, WI, USA), Raman spectrum (Micro Raman PL Mapping System, Dong Woo Optron Co., Kwangju, Korea), Atomic force microscope (AFM, NX10, Park Systems Co., Suwon, Korea), and Zetasizer (Malvern Instruments, Nano ZS, Worcestershire, UK) analyzes. FT-IR spectrum attenuated with total reflection mode was recorded for the sample (BPNSs) setting the wavelength range to 4000–500 cm^− 1^ at a resolution of 4.0 cm^− 1^. The topography of nanosheets was observed using respective AFM image, which was further analyzed using XEI software (Park Systems Co., Seoul, Korea). The average hydrodynamic size and zeta potential of nanosheets was determined using a Zetasizer.

### Cell culture

To determine the cytotoxicity of BPNSs in suspension, three different types of cells were investigated for the study. NIH3T3 fibroblast cells and nHDF normal human dermal primary cultured fibroblast cells were cultured in Dulbecco’s modified Eagle’s Medium (DMEM, Welgene, Daegu, Korea) with a high glucose concentration containing 10% fetal bovine serum (FBS, Welgene) and 1% antibiotic-antimycotic solution (Abs, Sigma-Aldrich., Saint Louis, MO, USA). HT1080 fibrosarcoma cells were cultured in Minimum Essential Medium (MEM, Welgene, Daegu, Korea) with 10% FBS and 1% Abs. The cells were stored in a humidified 96-well plate with a density of 5 × 10^3^ cells per well and incubated for 3 days. The cultured cells were treated with various concentrations of BPNSs (0–125 μg/mL) for 24 h and 48 h. After 24 h incubation period, the change in cellular morphology by the treatment of nanosheets was monitored using an optical microscope (Leica DMIL, Leica Microsystem, Wetzlar, Germany).

### Cytotoxicity of BPNSs

Measurement on cytotoxicity of BPNSs was carried out using two different assays, they are CCK-8 and LDH activity. For CCK-8 assay, CCK-8: DMEM (1:10) solution was dispensed into each well of cell plate containing the cells and samples. Absorbance was measured at 450 nm with ELISA Reader (SpectraMax 340, Molecular Device Co., Sunnyvale, CA) after incubation of solution mixture at 37 °C for 2 h 30 min in the dark. According to LDH assay, a 60 μL of reaction mixture containing catalyst (diaphorase/NAD+), dye solution of iodotetrazolium chloride and sodium lactate was transferred to the cell plate, which was preincubated with the cells and samples. Incubation was continued for 30 min at 25 °C in the dark and subsequently, absorbance was measured at 490 nm with ELISA Reader. The control was the cell line without the addition of samples (0 μg/mL). The experiment was repeated three times and the data were represented with standard deviation. Data were analyzed by one-way analysis of variance (ANOVA) followed by the Tukey test and significant difference compared to control was denoted at three levels using Origin 8 software analysis (*p < 0.05, **p < 0.01, ***p < 0.001).

## Results

### Spectral analyses

**Fig. 1:**
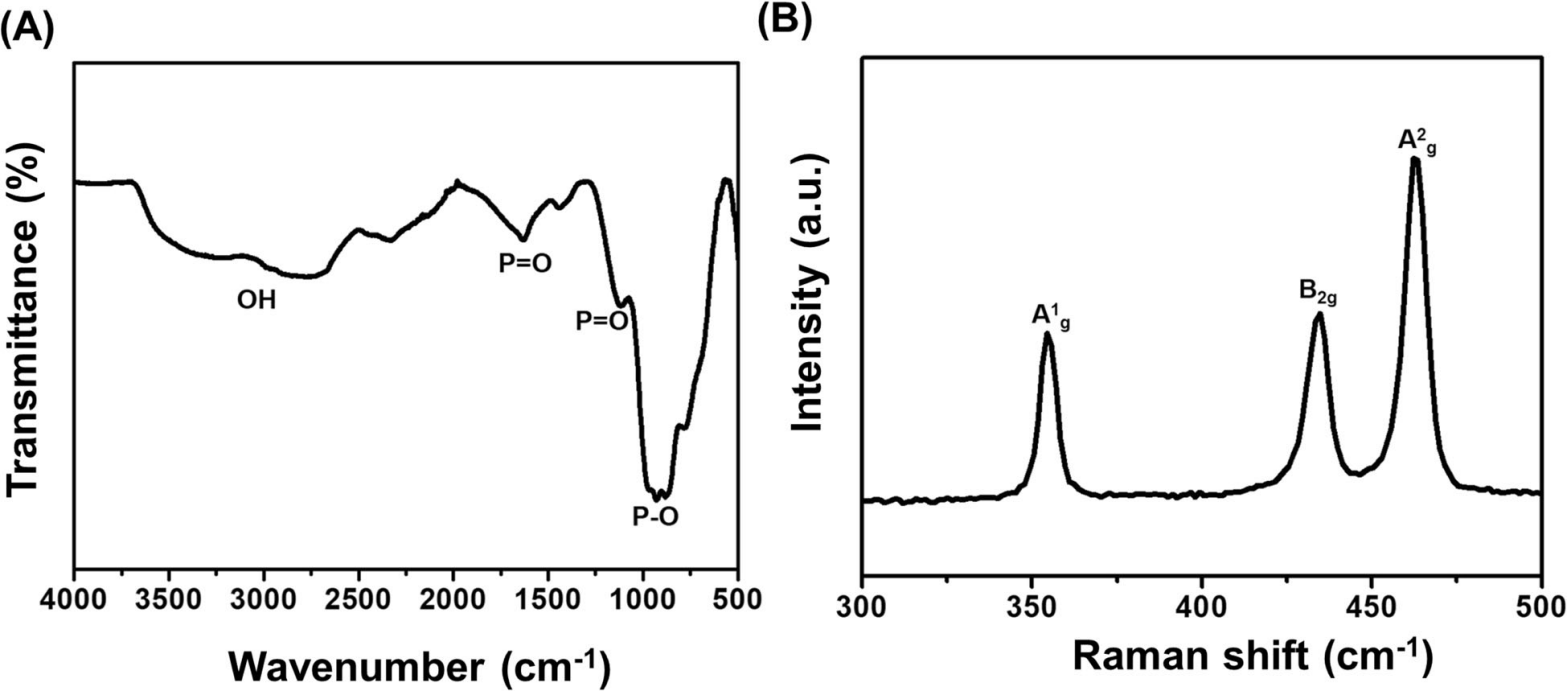
**a** FT-IR and **b** Raman spectrum of black phosphorus nanosheets (BPNSs)

FT-IR spectrum of BPNSs has displayed a stretching vibration of the P-O bond around 1000 cm^− 1^, as shown in Fig. 1a. The bands that appeared at 1100 cm^− 1^ to 1600 cm^− 1^ have been ascribed to the stretching vibration of the P=O bond. The bending and stretching vibrational modes of OH have been reflected at 2400 cm^− 1^ to 3000 cm^− 1^, respectively. According to Raman spectrum (Fig. 1b), the nanosheet has shown three prominent peaks at 354 cm^− 1^, 433^− 1^ and 462 cm^− 1^, which can be attributed to A^1^_g_ (out of plane mode), B_2g_, and A^2^_g_ (in-plane modes), respectively.

### Morphology of BPNSs

**Fig. 2:**
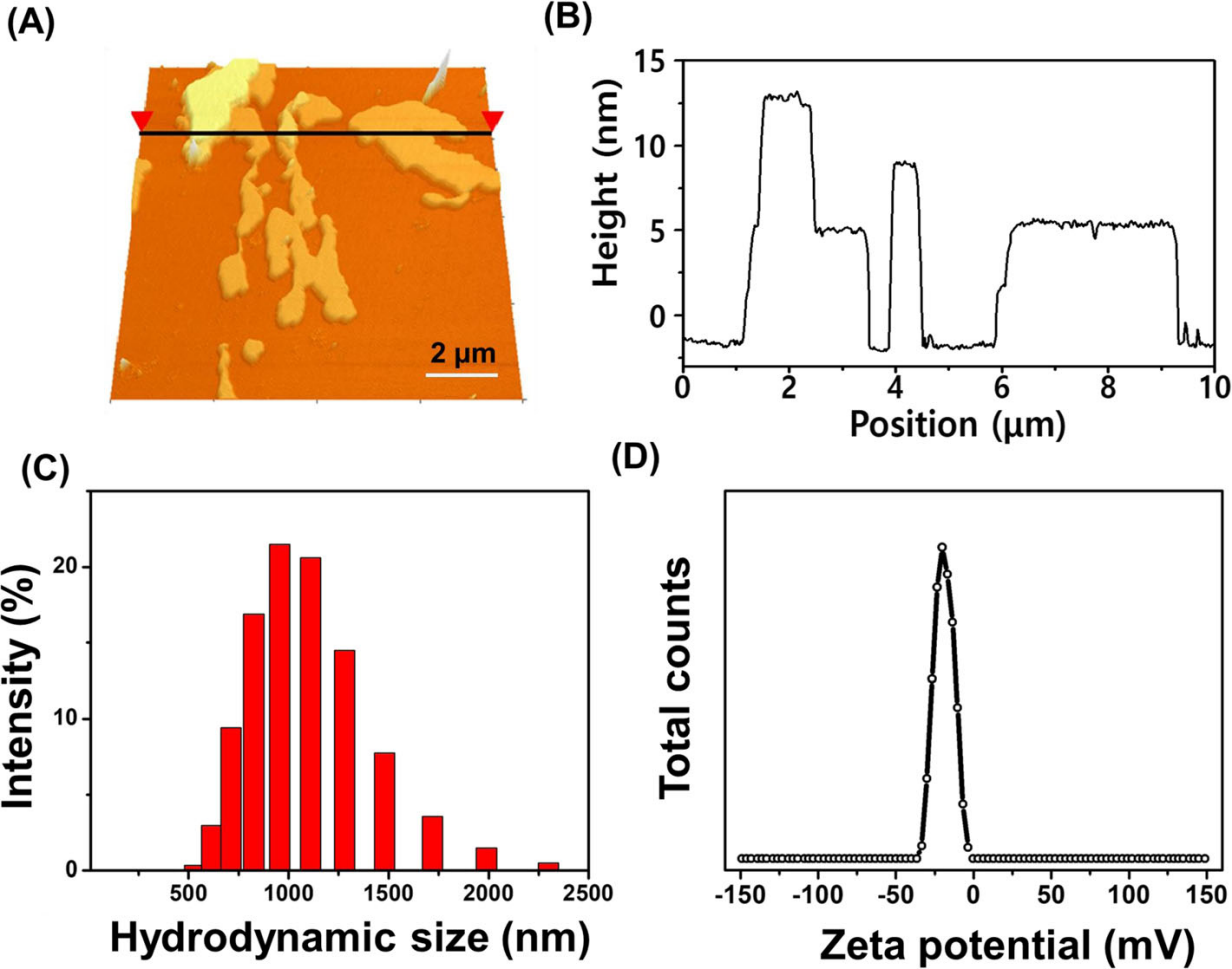
**a** AFM image and **b** corresponding height profile of BPNSs. The black linear line in **a** marks the position of nanosheets, height of which is measured. Particle Size distribution **c** and zeta potential **d** of nanosheets in solution

The morphology of the BPNSs has been observed from the AFM image as shown in Fig. 2a. The nanosheets appeared in aggregated form as the sample was air dried over the matrix. The corresponding height profile of BPNSs (Fig. 2b) describes that each aggregate has several layers ranging from 5.7–12.5 nm in size. Figure 2c and d reveal that hydrodynamic size and zeta potential of nanosheets have been 916 ± 185 nm and − 20 ± 4 mV, respectively. The polydispersion index value of nanosheets has been 0.7 ± 0.4.

### Cell viability

**Fig. 3:**
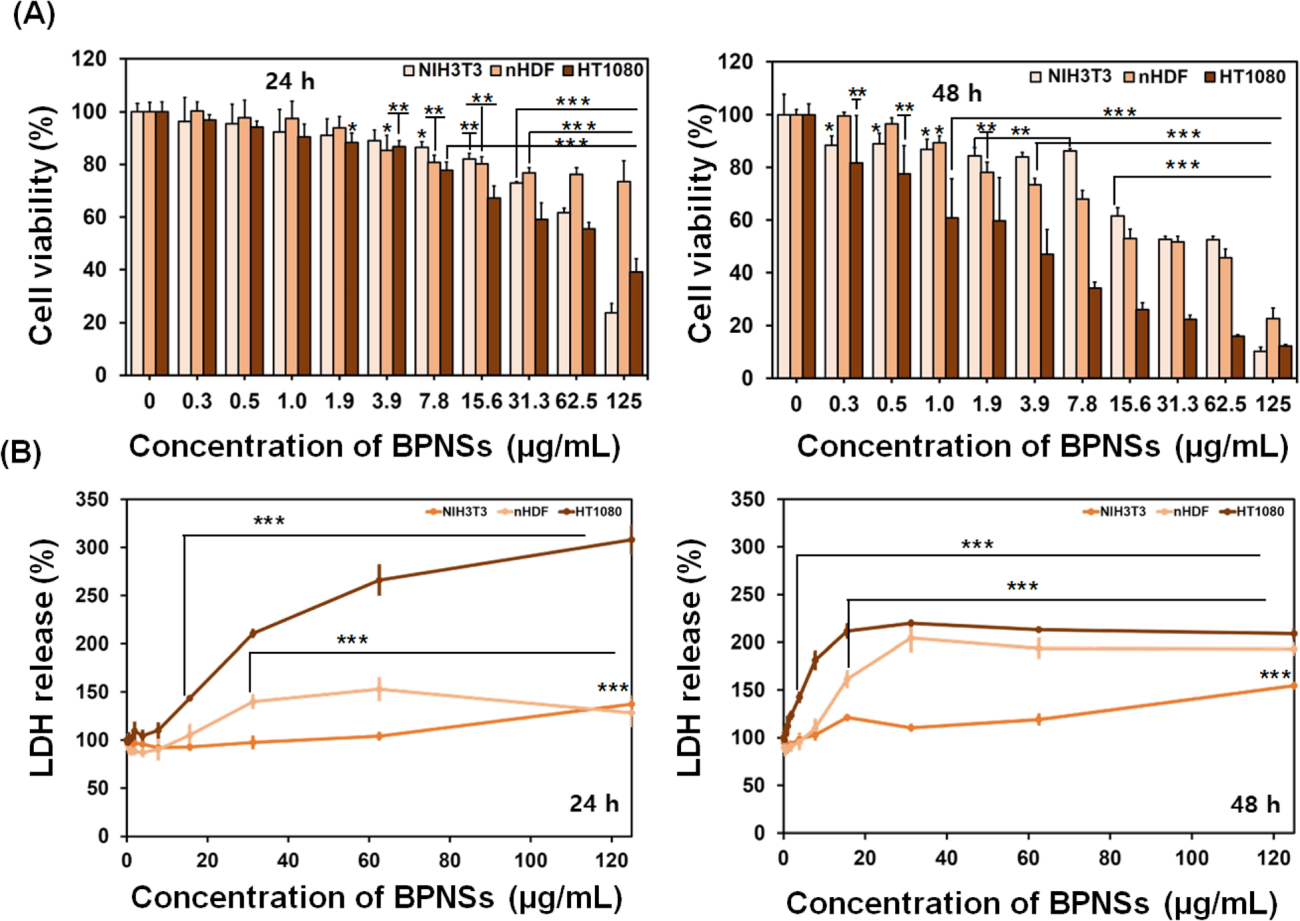
**a** Measurement of cell viability (%) of three different types of cells NIH3T3, nHDF and HT1080 determined by CCK-8 assay. The concentration range of nanosheets (BPNSs) is 0–125 μg/mL and the incubational time periods are 24 h and 48 h. **b** LDH release profile of the same. The control was the cell line without any additives (0 μg/mL). The data have been represented with standard deviation (*n* = 3; mean ± SD, ANOVA/Tukey’s-test; *P < 0.05, **P < 0.01, ***P < 0.001)

A decreasing trend in cell viability has been observed in a dose-dependent manner at different periods 24 h and 48 h when the fibroblast cells are treated with PBNSs, as shown in Fig. 3a. At 24 h of study, NIH3T3 and nHDF fibroblast cells have maintained cell viability up to the concentration of 15.6 μg/mL with more than 80% of viable cells. Fibrosarcoma cells (HT1080) have exhibited 78 ± 3% cell viability at 7.8 μg/mL. At 48 h of study, the cell viability of fibrosarcoma cells has been significantly affected when compared to normal fibroblasts (NIH3T3 and nHDF) even at low concentration of nanosheets (Fig. 3b). There was a significant difference in cell viability for fibrosarcoma cells when exposed to nanosheets at the concentration range of 0.3–125 μg/mL. The same toxic response has been reflected for NIH3T3 and nHDF cells at the concentration range of 15.6–125 μg/mL and 3.9–125 μg/mL of nanosheets, respectively.

All the cell lines investigated have shown concentration and time-dependent effects on lactate dehydrogenase activity, as shown in Fig. 3b. The low concentrated BPNSs (0–15.6 μg/mL) released a smaller number of LDH at 24 h of incubation with cells. The LDH level of NIH3T3 and nHDF fibroblast cells, at 62.5 μg/mL of BPNSs, had increased by 3 and 50%, respectively, and that of HT1080 fibrosarcoma cells increased by more than 100% at 31.3 μg/mL. It has been observed that the amount of the initial LDH release of nHDF fibroblasts and HT1080 fibrosarcoma cells have increased significantly than that of NIH3T3 at 48 h of study. However, there was a steadiness in the amount of LDH released for all types of cells when the concentration of nanosheets increases above 31.3 μg/mL.

**Fig. 4:**
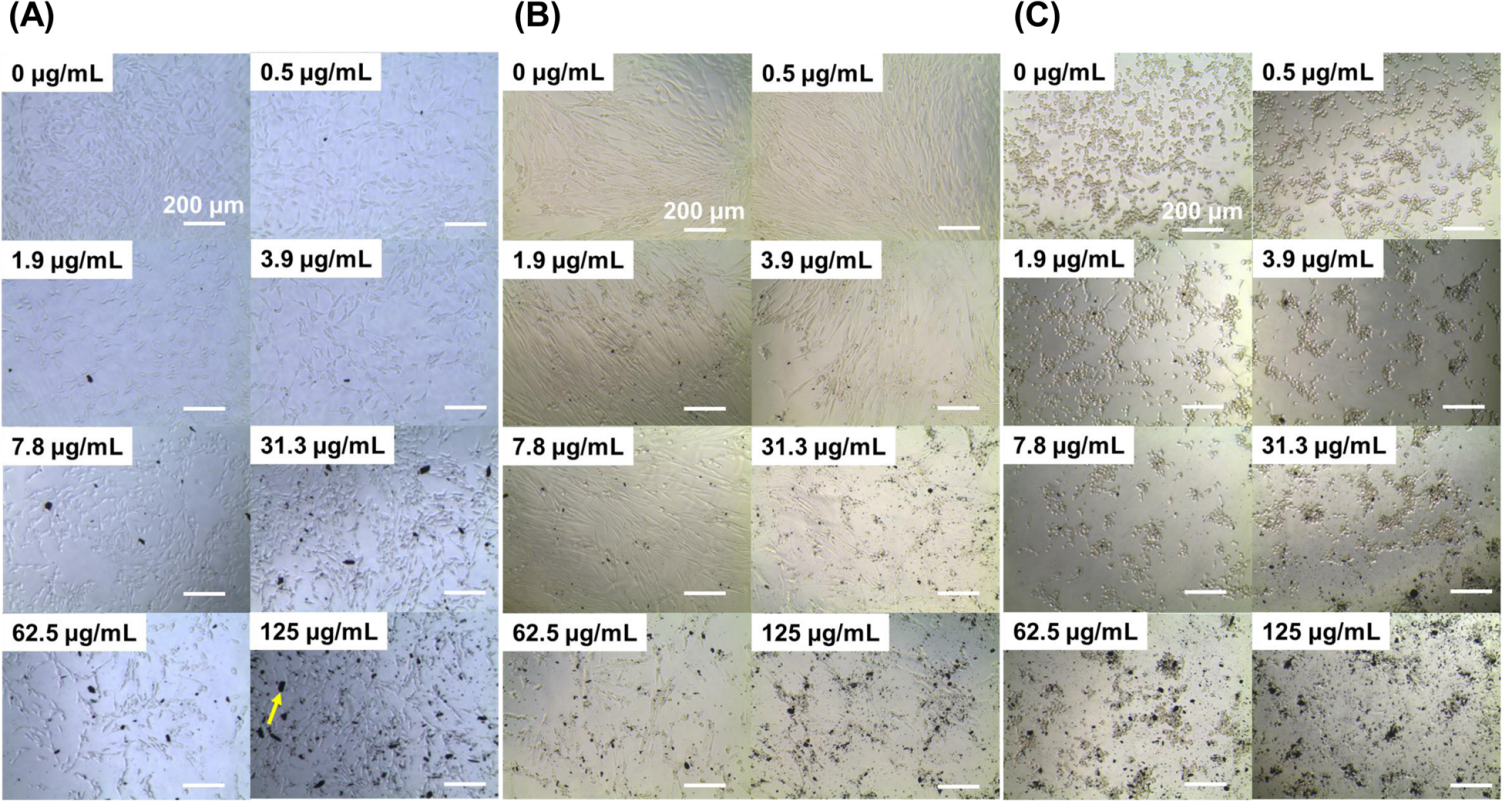
Optical microscope images of **a** NIH3T3 fibroblast, **b** nHDF fibroblast, and **c** HT1080 fibrosarcoma cells cultured with BPNSs (0–125 μg/mL) after 24 h. The control was the cell line without any additives (0 μg/mL). The scale bar represents 200 μm. The arrow indicates aggregated nanosheets outside the cell

The effect of BPNSs on cellular morphology has been observed using optical microscope images at 24 h incubation of nanosheets with the cells (Fig. 4a-c). The nanosheets that do not permeate the cells have been found aggregated outside the cells. As the concentration of nanosheets increases, there were no changes in the morphology of NIH3T3 cells. At the same time, nHDF and HT1080 cells have shown a decreased cell count at and above 31.3 μg/mL and 7.8 μg/mL, respectively.

## Discussion

The respective functional groups of BPNSs have been confirmed from the spectral analyses of FTIR and Raman spectrum. The results were in close agreement with the previously published literature [13–15]. AFM result has informed that the BPNSs produced from liquid exfoliation procedure have contained several layers with different sizes. The Zetasizer analyses revealed that BPNSs have larger hydrodynamic size along with higher polydispersion index, which showed that the nanosheets are agglomerated in solution. However, the average zeta potential value indicates that the nanosheets are moderately stable against further aggregation in solution. The negative sign in zeta potential is due to the P-O bond on the surface of nanosheets. It is important to note that the interaction of protein and cell with nanoparticles is majorly dependent on the size and charge of the nanoparticles [16]. The negatively charged nanoparticles have been reported to be preferable for clinical use as they would have longer blood circulation life evading interaction with similarly charged serum proteins [17].

Three different fibroblast cells are investigated to determine cell viability exposing to BPNSs. NIH3T3 and nHDF are normal healthy fibroblast cells whereas HT1080 is a cancerous fibroblast cell line. The normal fibroblast cells are also chosen from different animal sources mouse (NIH3T3) and human (nHDF). Skin and lung are the majorly affected organs for those who work in the places of nanoparticle exposure for prolonged time. We have selected fibroblast cells for the in vitro cytotoxicity studies because they are heterogeneous and dynamic cell lineage presenting with different populations between human tissues [18, 19]. Fibroblast cells can produce extracellular matrix and collagen, which play critical roles in wound healing [20]. Cancerous cells have been found more sensitive towards BPNSs exposure than normal cells, which is evident from cell viability assays. The order of time and concentration-dependent toxic response has been observed as follows, HT1080> nHDF> NIH3T3. CCK-8 assay was carried out to evaluate cell viability through the metabolic activity of mitochondria.

When the cell membrane is damaged, the enzyme LDH present in the cytoplasm is released to the external medium and hence cytotoxicity of BP particles is correspondent with the disruption of cell membrane [21]. LDH release from the cells nHDF and HT1080 are greater than that of NIH3T3 at 24 h of the study. Moreover, LDH release from NIH3T3 has been significantly less than that of nHDF and HT1080 at 48 h of the study, which corroborates with the results of cell viability. As far as cell morphology and cell count are concerned, the optical microscope images describe that morphology of the investigated cells are not affected but the cell counts have been found reduced when exposed to higher concentrated BPNSs (≥31.3 μg/mL). As BPNSs affect cellular viability in cancerous fibroblast cells than normal cells in a time and dose-dependent manner, the present work can be further processed to cancer treatment and for in vivo animal studies.

## Conclusions

Black phosphorus nanosheets synthesized using liquid exfoliation procedure were characterized with appropriate physicochemical properties such as identification of functional groups, hydrodynamic size, and zeta potential of the nanosheets. The cytotoxicity of BPNSs has been investigated subjecting different fibroblast cell lines viz. NIH3T3, nHDF, and HT1080. The preliminary study exemplifies that BPNSs has exhibited different level of toxicity depending on cell line type, exposure time and concentration. The salient findings of the work reveal that the nanosheets are more toxic to cancerous HT 1080 cells when compared to its toxic response to normal cells. We conclude that a comprehensive study is still required to elucidate the biological activities of BPNSs through in vitro and in vivo analyses before considering the compound for its biomedical applications including cancer treatment in the future.

## Data Availability

All data generated and analyzed in this study are available from the corresponding author on request.

## Abbreviations

AFM: Atomic Force Microscope
BPNSs: Black phosphorus nanosheets
CCK-8: Cell counting kit-8
FT-IR: Fourier Transform Infrared
LDH: Lactate dehydrogenase
nHDF: Primary human dermal fibroblasts
